# An n-of-1 Trial Service in Clinical Practice: Testing the Effectiveness of Liuwei Dihuang Decoction for Kidney-Yin Deficiency Syndrome

**DOI:** 10.1155/2013/827915

**Published:** 2013-09-23

**Authors:** Huang Yuhong, Liu Qian, Liu Yu, Zhao Yingqiang, Li Yanfen, Yu Shujing, Qin Shufang, Sun Lanjun, Zou Shuxuan, Wang Baohe

**Affiliations:** ^1^Second Affiliated Hospital, Tianjin University of Traditional Chinese Medicine, Tianjin, China; ^2^Tianjin University of Traditional Chinese Medicine, Tianjin, China

## Abstract

*Objective*. To describe the clinical use of n-of-1 RCTs for kidney-Yin deficiency syndrome that is a traditional Chinese medicine syndrome in publicly clinical practice in China. *Methods*. Our study included patients with kidney-Yin deficiency syndrome, using a within-patient, randomized, double-blind, crossover comparison of Liuwei Dihuang decoction versus placebo. *Outcome Measures*. Primary outcome measures included number of individual completion rates, response rate, and post-n-of-1 RCTs decisions. Secondary measures were the whole group score of individual Likert scale, SF-36 questionnaire. *Results*. Fifty patients were recruited and 3 were not completed. Forty-seven patients completed 3 pairs of periods, 3 (6.38%) were responders, 28 (59.57%) were nonresponders, and 16 (34.05%) were possible responders. Doctors and patients used the trial results to making decision. Three responders stayed on the medication management, 28 nonresponders ceased the LDD, 7 patients of the 16 possible responders could not give clear decision, and the others kept the same medication station. Among the whole group, neither the individual Likert score nor the SF-36 showed any statistical differences between LDD and placebo. *Discussion*. More attention should be paid to choose experienced TCM doctor as investigator and keep the simulant same with test medication in n-of-1 RCTs of TCM and sufficiently biological half-life period of Chinese medicine compound.

## 1. Introduction

Liuwei Dihuang decoction (LDD) was first recorded in the *Knack of Prescription in Pediatrics* (xiao'er yaozheng zhijue) in AD1114 of the Beisong Dynasty. Its author is Qian Yi, who was famous pediatrician and prescribed the LDD to dysplasia in children. From the Chinese medicine perspective, dysplasia in children is thought to be associated with a decline in kidney Yin. Hereafter, the LDD had been practiced in many diseases except the dysplasia in children. Until the Qing Dynasty (AD1644–1911), in the *comprehension of medicine* (yixue xinwu) that records the practical and concise experience of the famous doctor Zhongling Cheng, the LDD had been reported to be practiced in less than 20 diseases, such as stroke, headache, urinary incontinence, lumbago, and asthma. From Beisong Dynasty to Qing Dynasty, the LDD had been practiced not only in pediatrics but also in immune, endocrine, digestive, respiratory, urinary, and circulatory system diseases [1].

The Liuwei Dihuang decoction, a classic Chinese medicinal formula, is a compound prescription comprising six ingredients—Rehmanniae Radix Praeparata, Dioscoreae Rhizoma, Corni Fructus, Poria, Moutan Cortex, and Alisma Rhizoma. It has been reported that it effectively inhibits the development of benign prostatic hyperplasia [2]; decreases proteinuria, protects kidney function, and ameliorates histopathology [3]; alleviates *β*-amyloid-induced toxicity [4]; lowers body weight and improves insulin and leptin sensitivity [5]; and so on. Based on gene and phenotype information associated with both LWDH (Liuwei Dihuang) herbs and LWDH-treated disease, LWDH-treated diseases show high phenotype similarity and identified certain “comodules” enriched in cancer pathways and neuroendocrine-immune pathways, which may be responsible for the action of treating different diseases by the same LWDH formula [6].

At present, in clinical practice, LDD has been used to improve or restore declined functions related to aging and geriatric disease such as impaired mobility, vision, hearing, cognition and memory [4]. Its indication is deficiency of Kidney Yin including many diseases, how to establish evidence to guide doctor and patient to administer LDD rationally is considered. The n-of-1 trials maybe are a promising method. Nikles et al. had developed n-of-1 trials into clinical tests for several conditions, designing them to assist clinicians to identify whether a specific patient responds to a particular drug for chronic stable conditions in which individual response to treatment is variable [7]. We performed n-of-1 randomized controlled trials (n-of-1 RCTs) of LDP whose indication is deficiency of kidney-Yin in order to guide the clinicians and patients whether to take it continually.

## 2. Methods

### 2.1. Medicine Choose

There are many forms of the LDD on sale in China, such as honey bolus, water pill, liquid, and soft capsule. In this study, we choose the LDD soft capsule that is best to make simulant than other forms because of the special flavor and color of Chinese herbs.

### 2.2. The Clinical n-of-1 Service

The trial was conducted in the second hospital affiliated to the Tianjin University of TCM in China from September 2009 to September 2011. We supplied request and consent forms to interested doctors. Doctors could explain the process to interested patients and obtained informed consent. The Kanion Pharmaceutical Co., Ltd, standing in middle-east of china Jiangsu Province that was approved to produce LDD (soft capsule) in November 2000 by state food and drug administration of china (SFDA) prepared the study medication and randomized it according to a computer-generated randomization schedule. Kits containing the correct sequence of randomized medicine were posted to our hospital. A research pharmacist at the hospital received the study medicines labeled with treatment identification and distributed them to eligible participants sequentially according to the randomization schedule. 

### 2.3. Patients

The trial protocol was approved by the ethics committees of the Second Affiliated Hospital of Tianjin University of Traditional Chinese Medicine in February 2009. Informed written consent was given before the trial began and the participants were free to withdraw at any time during the study.

Participants were eligible if they had a clinical diagnosis of deficiency of kidney Yin (Table 1) according to the same diagnosis of two senior TCM clinicians who assessed separately, were aged 25 to 65 years, and had uncertainty about treatment effectiveness of LDP. The exclusion criteria were (1) major neuropsychiatric disorder (schizophrenia, epilepsy, alcohol abuse, anorexia, and so forth); (2) planning to have a baby; (3) cardiocerebral vascular diseases, insufficiently controlled hypertension or hypotension, thromboembolic diseases, gastrointestinal diseases affecting drug absorption, hematopoietic system diseases, or autoimmune system diseases; (4) abnormal liver function or abnormal renal function; (5) others that the investigator assessed not suitable to take part in study. Participants came from the Second Affiliated Hospital of Tianjin University of Traditional Chinese Medicine.

### 2.4. Randomization

**Figure 1:**
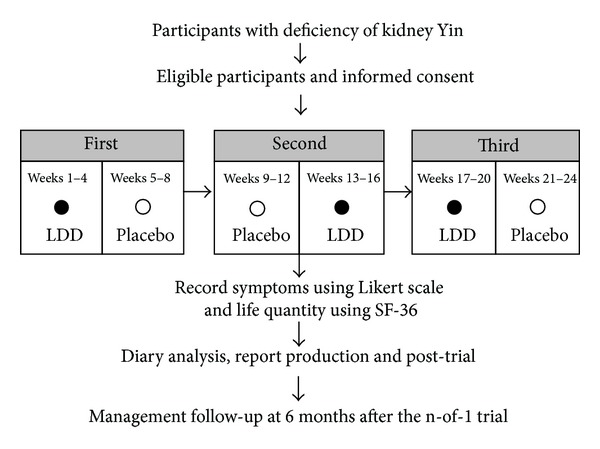
Flowchart for the n-of-1 trial. In this example, “weeks 1–4” is a treatment period. Weeks 1–4 and weeks 5–8, combined, are the first pair of treatment periods. The whole n-of-1 trial consists of the 3 pairs of treatment periods.

The individual studies ran for 24 weeks. Patients undertook 3 pairs of 8-week treatment periods (each period consisting of 4-week LDD and 4-week placebo in random order). Each pair contained the LDD and the placebo, two treatment periods (Figure 1). Both the patients and doctors interacting with them and the research assistant were all blind to when patients were taking the LDD or the placebo.

### 2.5. Data Collection

Patients completed diaries containing ten clinical kidney-Yin deficiency syndromes rated by the Likert scale (strongly agree 5, agree 4, neither agree, or disagree 3, disagree 2, strongly disagree 1) during and at the end of each treatment period, which was used to assess the degree of kidney-Yin deficiency before and after treatment. Items of the Likert scale of Kidney-Yin deficiency syndrome were selected from TCM teaching material issued by Chinese government and were discussed by 5 senior TCM clinical doctors before validity and reliability tests. The validity and reliability of the Likert scale was tested in a small amount of people (15 patients with kidney-Yin deficiency and 20 cases of healthy college students) before research. The survey results show that it has good discriminant validity.

Patients also completed the SF-36 (the MOS item short from health survey, SF-36) at the end of each treatment period, which were used to measure perceived health and quality of life.

We collected information about medication history, demographic variables, and treatment decision immediately after the n-of-1 trial.

### 2.6. Data Analysis

An n-of-1 RCT was considered “responder” if Likert scale score and SF-36 score were both more favorable response to LDD treatment (in all 3 treatment pairs), “possible responder” (in 2 of 3 treatment pairs), or “nonresponder.”

To address the effect of LDD on the entire group, we conducted repeated-measures analysis of variance, examining the effects of treatment, pair, and the treatment-pair interaction. Besides, we pooled the standardized mean differences from every patient who completed the trial by using meta-analysis, comparing the effect of LDD with that of placebo.

All statistical analyses, except for the meta-analysis, were performed using SPSS 13.0. The meta-analysis was calculated using RevMan5.0. All descriptive data are expressed as means and standard deviations. All estimates are expressed as mean differences and 95% CI unless otherwise stated.

## 3. Results

### 3.1. Patients

Six doctors in the Second Affiliated Hospital of the Tianjin University of Traditional Chinese Medicine participated in this study. Fifty patients who met trial entry criteria were recruited, and 47 people completed the trial. The reasons for the three withdrawals were that 1 because of right oophorectomy, 1 because of leg injury, and 1 because of noeffectiveness.  Table 2 presents the characteristics of the 47 patients who completed the study.

### 3.2. Individual n-of-1 RCTs

**Figure 2:**
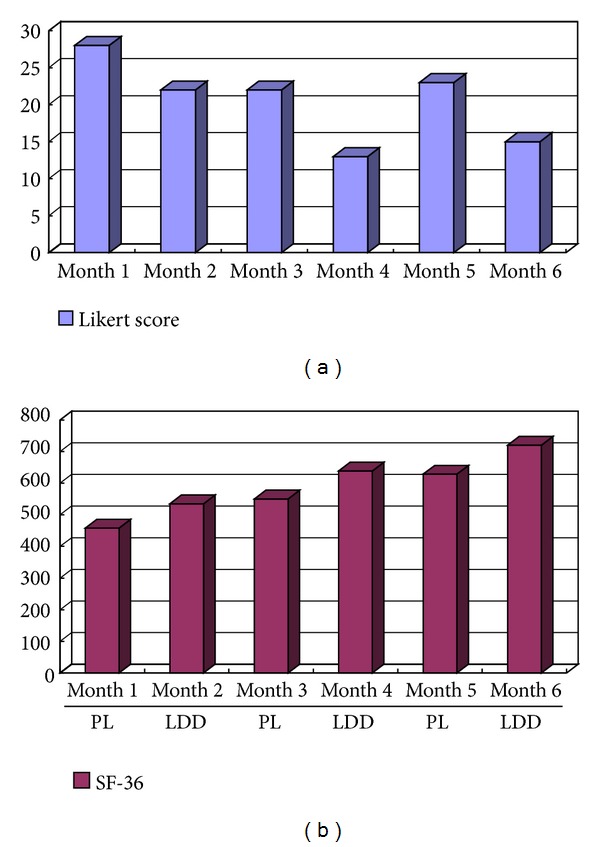
Example of a responder to LDD. The responder's Likert score and SF-36 show clear differences between months 1, 3, and 5 (placebo) and months 2, 4, and 6 (LDD). Higher scores of Likert score indicate worse behavior. Higher scores of SF-36 indicate better behavior.

Three patients met the criteria for a responder, 16 patients met the criteria for a possible responder, 28 patients were non-responders, and no patients met criteria for responder to placebo.  Figure 2 presents details of one clear responder.

The n-of-1 trial had a marked effect on management. Medication immediately after trial changed for 29 (65.91%) of 47 of those completing the trial: of 41 patients who had taken the LDD, 26 patients ceased it, 8 patients kept the same medication station, and 7 patients could not give clear decision. Of 6 patients who had not taken the LDD, 3 patients began to take it, 2 had no plan to take it, and 1 could not give clear decision.

It is necessary to put the post-n-of-1 trial management decisions into context. Doctors and patients used the trial results for making decision. Three responders stayed on the medication management, 28 non-responders ceased the LDD, 7 patients of the 16 possible responders could not give clear decision, and the others kept the same medication station (Table 3).

### 3.3. Group n-of-1 RCTs

Results from repeated-measures analysis show individual Likert score improved over time, but no significant differences between LDD and placebo group (Table 4). Although there was an improvement over time for every SF-36 domain (*P* < 0.05), except for SF-36 physiology and SF-36 emotion, these effects were unrelated to LDD being used, nor any interaction between treatment and pair for any of the SF-36 domain (Table 5).

**Figure 3:**
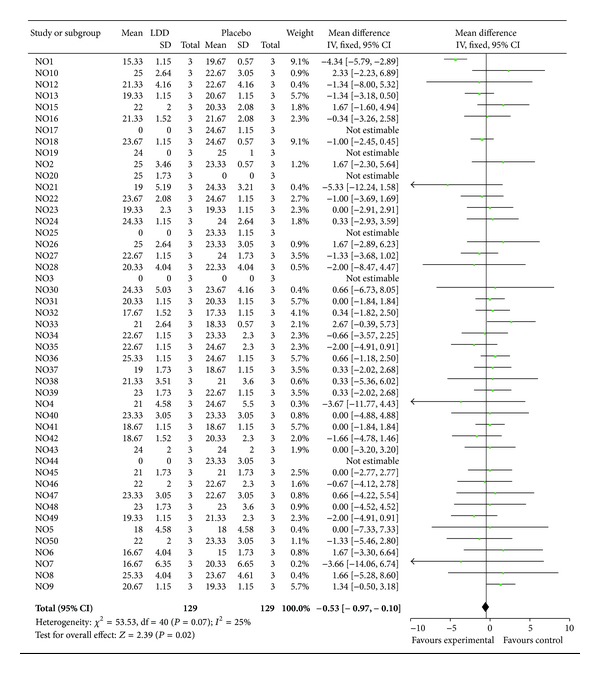
Meta-analysis of individual Likert score between LDD and placebo.

**Figure 4:**
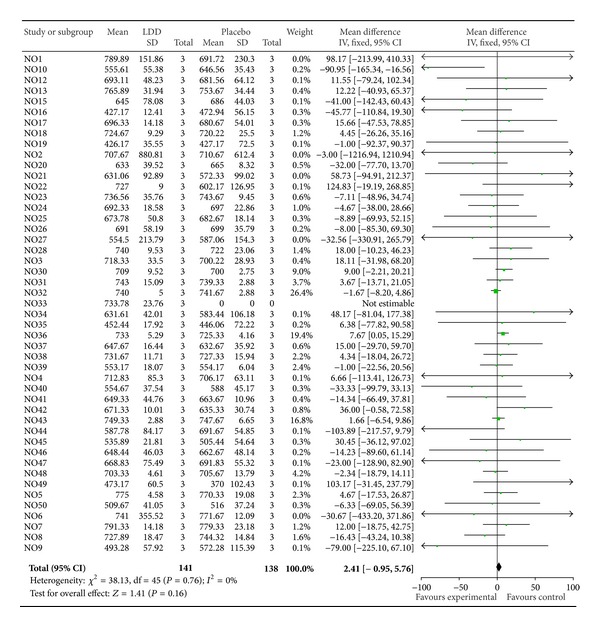
Meta-analysis of SF-36 total score between LDD and placebo.

Patients show same level of individual Likert score between LDD and placebo in meta-analysis. The standardized mean difference in the Likert score between LDD and placebo was −0.09 (95% CI −0.36 to 0.17), using a weighted mean difference technique. Because of no heterogeneity in results across the patients (test for heterogeneity, *P* = 0.07), fixed model was used (Figure 3). Similarly, patients show no difference of SF-36 total score between LDD and placebo. Overall, the standardized mean difference SF-36 total score between LDD and placebo was 2.41 (95% CI −0.95 to 5.76), using a weighted mean difference technique. Because of no heterogeneity in results across the patients (test for heterogeneity, *P* = 0.76), fixed model was used (Figure 4).

Adverse effects were not found between LDD and placebo.

## 4. Discussion

### 4.1. Research Background

Traditional Chinese medicine (TCM) whose basic principle of treatment is the syndrome differentiation-based treatment emphasizes the individualized medicine. The n-of-1 trial may be a good means to guide clinical practitioner and patient to manage the TCM. LDD is one of the most classical formulas, which is OTC medicine in China, and is consumed numerously every year. Abuse of LDD also may cause some side effects such as indigestion and loose stool [1]. The n-of-1 trial is an effective means to decrease the abuse of LDD. Our study is a good example.

### 4.2. Summary of Main Findings

The n-of-1 design is an attractive technique to define efficacy on an individual basis. In our study, the n-of-1 trial identified 3 patients for whom LDD was clearly beneficial, 16 who showed nonsignificant benefit and 28 who showed no benefit. Despite its weak extrapolation, we compare the difference between LDD and placebo. Group results showed no apparent effect of LDD on individual Likert score or any of eight domains of the SF-36.

### 4.3. Strengths and Weaknesses of the Study

The n-of-1 clinical trial can leverage study design and statistical techniques associated with standard population-based clinical trials, including randomization, washout and crossover periods, and placebo controls. 

Many Chinese are confident with the TCM and desired to choose TCM for treatment of chronic diseases or health care, so in our study, the patient compliance is good. A limitation of the trial was washout period which has not been fully considered. Because it is difficult to know biological half-life period of Chinese medicine compounds, we speculate that 2 days of biological half-life period in our study may not be enough, which resulted in residual effects of traditional Chinese medicine interfered the differences between LDD and placebo. It in part explains why LDD showed non-significant benefit compared with placebo in our study.

### 4.4. Relationship of Our Study to the Existing Literature

While not observed in our study, the documented adverse effects of LDP are reported in other articles, such as diarrhea and inappetence [1]. Although to date, no serious adverse reactions have been reported about LDD, it is still necessary to pay attention to the abuse of LDD.

The fundamentality of TCM is syndrome differentiation and treatment. In our study, all the patients were included based on syndrome differentiation and had a clinical diagnosis of deficiency of Kidney Yin. According to the theory of traditional Chinese medicine, which deems that sideeffects of Chinese medicine will not appear as long as syndrome differentiation is accurate, we guess that syndrome differentiation in our study is accurate. That could explain why the drug's side effects are not found in our study.

 The n-of-1 or single subject clinical trial considers an individual patient as the sole unit of observation in a study investigating the efficacy or side-effect of different interventions. The ultimate goal of an n-of-1 trial is to determine the optimal or best intervention for an individual patient using objective data-driven criteria. So, extrapolation of n-of-1 clinical trial will be weak. Unlike previously reported studies [8] which were used to treat diabetes when combined with antidiabetic and applied blood sugar as therapeutic effect index, our study used kidney-Yin deficiency syndrome and health-scale and the group results showed no trend in favor of LDD being of benefit.

### 4.5. Implications from the Study

Despite obvious appeal and wide use in educational settings of n-of-1 trial, it has been used sparingly in medical and general clinical settings [9]. We can only speculate why. Perhaps we only slightly penetrated doctors' awareness; the process requires considerable input from doctors [7]. We briefly reviewed the literatures and found only one n-of-1 clinical trials report about botanical that is the spirulina to treat chronic fatigue in four n-of-1 randomized controlled trials [10].

Traditional Chinese medicine holds a large market in China. Many people seek health care treatment using traditional Chinese medicine. Some TCM compound prescriptions benefit some symptoms such as cough and frequent urination. The primary purpose of this study is to restrict the abuse of Chinese patent drugs, but due to the characteristics of differentiation-based treatment in TCM, it is not appropriate to evaluate these compound prescription effects in population-based clinical trials that may not be resolve the question of clinical equipoise in these clinical settings because these symptoms exist in many diseases. It has been suggested that the main role of n-of-1 trials in clinical practice is to cancel useless treatment rather than advocate drug treatment [11]. The n-of-1 trial may be a promising approach that essentially starts out small and focused and then works its way towards insights that would prevent unnecessary use of Chinese patent drugs. 

In summary, this study does not support the general application of LDD for patients with deficiency of kidney Yin. Our data also suggest that more attention should be paid to choose experienced TCM doctor as investigator and keep the simulant same with test medication in n-of-1 trial of TCM and sufficiently biological half-life period of Chinese medicine compound.

## Figures and Tables

**Table 1:** Diagnosis criteria of deficiency of kidney-Yin.

(1) Dizziness
(2) Tinnitus
(3) Flaccid waist and knees
(4) Hectic fever
(5) Dry mouth and throat
(6) Night sweat
(7) Spermatorrhea
(8) Thirst and drink
(9) Red tongue and less fur
(10) Thready and rapid pulse

Patients who have not less than 3 items above can be diagnosed as deficiency of kidney-Yin.

**Table 2:** Patient characteristics.

Characteristics	
Age, yr	48.76 ± 2.01
Females/males, *n*	35/15
Married/unmarried, *n*	49/1
Taken the LDD previously, *n*	41
Baseline individual Likert score	15.23 ± 3.91
Baseline SF-36 score	118.44 ± 13.58

Values are absolute numbers or mean ± SD.

**Table 3:** Management changes immediately post-n-of-1 trial.

	Taking LDD	No taking LDD	Unclear decision	Total
Patients had taken LDP or not, *n* (%)				
Yes	8 (19.51)	26 (63.42)	7 (17.07)	41 (100)
No	3 (50.00)	2 (33.33)	1 (16.67)	6 (100)
Total	**11 (23.40)**	**28 (59.58)**	**8 (17.02)**	**47 (100)**

The n-of-1 trials, *n* (%)				
Responders	3 (100.00)	0 (0.00)	0 (0.00)	3 (100)
Possible responders	9 (56.25)	0 (0.00)	7 (43.75)	16 (100)
Nonresponders	0 (0.00)	28 (100.00)	0 (0.00)	28 (100)
Total	**12 (25.53)**	**28 (59.57)**	**7 (14.90)**	**47 (100)**

**Table 4:** Repeated measure of individual Likert score.

Source	Sum of squares	df	Mean square	*F*	*P*
Group	5.121	1	5.121	0.267	0.607
Pair	329.199	2	164.599	30.478	0.000
Group ∗ pair	2.433	2	1.216	0.225	0.789
Error (group)	1766.681	92	19.203		
Error (pair)	993.702	184	5.401		

Test statistics of Mauchly's test of sphericity: *W=0.954*, *P=0.117*. Individual Likert score improved significantly in different pair. No significant differences between LDD and placebo. No interaction between group and pair.

**Table 5:** Repeated measure of SF-36 total score.

Source	Sum of squares	df	Mean square	*F*	*P*
Group	10.150	1	10.150	0.000	0.985
Pair	60248.979	1.775	33936.371	10.660	0.000
Group ∗ pair	3261.686	1.775	1837.206	0.577	0.543
Error (group)	2668184.506	92	29002.005		
Error (pair)	519956.205	163.332	3183.425		

Test statistics of Mauchly's test of sphericity: *W=0.873*, *P=0.002*. Greenhouse-Geisser was used to adjust df. SF-36 total score improved significantly at different pair. No significant differences between LDD and placebo. No interaction between group and pair.
